# Description of a novel mating plug mechanism in spiders and the description of the new species *Maeota setastrobilaris* (Araneae, Salticidae)

**DOI:** 10.3897/zookeys.509.9711

**Published:** 2015-06-22

**Authors:** Uriel Garcilazo-Cruz, Fernando Alvarez-Padilla

**Affiliations:** 1 Laboratorio de Aracnología. Facultad de Ciencias, Universidad Nacional Autonoma de Mexico s/n Ciudad Universitaria, México D. F. Del. Coyoacán, Código postal 04510, México

**Keywords:** Reproduction, Neotropics, taxonomy, sperm priority

## Abstract

Reproduction in arthropods is an interesting area of research where intrasexual and intersexual mechanisms have evolved structures with several functions. The mating plugs usually produced by males are good examples of these structures where the main function is to obstruct the female genitalia against new sperm depositions. In spiders several types of mating plugs have been documented, the most common ones include solidified secretions, parts of the bulb or in some extraordinary cases the mutilation of the entire palpal bulb. Here, we describe the first case of modified setae, which are located on the cymbial dorsal base, used directly as a mating plug for the Order Araneae in the species *Maeota setastrobilaris* **sp. n.** In addition the taxonomic description of *Maeota setastrobilaris* **sp. n.** is provided and based on our findings the geographic distribution of this genus is extended to the Northern hemisphere.

## Introduction

Arthropod reproductive systems are an interesting topic of research where several mechanisms of sexual selection have evolved. In this context females seek to maximize sperm diversity throughout polyandry, while males try to maximize parenthood by preventing the female from being inseminated by other males. One possibility of preventing subsequent inseminations is the production of mating plugs which might be a result of an arms race or male-male competition (Eberhard 1985; Eberhard and Huber 2010), or as part of sperm competition (Uhl et al. 2010). In any case, mating plugs are perfect examples of structures that block the female copulatory ducts (Eberhard 1985) regardless of the hypotheses that have been postulated to explain their evolution.

Production of mating plugs in Araneae have mainly evolved in Entelegynae reproductive systems characterized by the presence of two different pairs of ducts, one specialized for insemination and the other for fertilization. Mating plugs are only found on the copulatory duct openings and have been classified as defensive traits to avoid sperm competition, in contrast to offensive traits that introduce secretions in the female genital track detering the quality of previously deposited sperm. Both mechanism were explained by the sperm competition hypothesis; however, alternative non-exclusive functions under natural selection such as retaining sperm in an advantageous position for fertilization, preventing sperm leakage or desecration have been postulated (Uhl et al. 2010).

Mating plugs can be either chemical or physical. The former are secretions produced by structures such as bulb glands, the spermatic duct, glands in the male mouth area or plugs produced by secretion of female origin (Eberhard 1983; Uhl et al. 2010). Plugs in zodariid spiders have even been attributed to specialized setae on the dorsal area of the cymbium, i.e., in *Storenomorpha reinholdae* Jocqué & Bosmans, 1989 and *Cicynethus* sp. (Jocqué 1991), but thorough documentation of these cases are still in progress as more evidence is adquired. Plugs may also be formed by male genital structures like the embolus, embolus and conductor, paracymbium, and there are extraordinary cases where the complete palpal bulbs or even the whole male (*Tidarren* spp.) are used as plugs after insemination (Uhl et al. 2010). Detachment of tibial setae has been suggested in Paccius cf. scharffi Platnick, 2000; although this observation has not been thoroughly tested (Uhl et al. 2010).

The genus *Maeota* Simon, 1901 has currently four described species inhabiting South America: *Maeota dichrura* Simon, 1901, *Maeota dorsalis* Zhang & Maddison, 2012, *Maeota flava* Zhang & Maddison, 2012 and *Maeota simoni* Zhang & Maddison, 2012 (World Spider Catalog 2015). The monophyly of this genus is currently supported only by molecular evidence (Zhang and Maddison 2012). These authors provided a diagnosis for *Maeota*, comparing it to the known diversity of other Euophryinae, as the following combination of features: medium small-sized spiders (ca. 2.7–4.3 mm), chelicerae with two promarginal and one retromarginal teeth, first tibia with three pairs of ventral macrosetae and first metatarsus with two pairs, plane of the embolic spiral more or less perpendicular to the longitudinal axis of the bulb and tegulum with a proximal lobe (Zhang and Maddison 2012). This diagnosis may change as more *Maeota* species are discovered and these diagnostic character hypotheses tested.

The present study describes the new species *Maeota setastrobilaris* sp. n., which possesses a new kind of physical mating plug mechanism in the Order Araneae, which functions by the detachment of modified cymbial setae, referred hereafter as strobilate setae.

## Methods

The specimens here described were collected as part of a spider inventory in a tropical forest remnant located near the city of Xilitla from August 2011 to June 2012. This inventory was made following standardized protocols (Coddington et al. 1991). All specimens were collected inside a one hectare plot (with central coordinates 21°23'50.9"N, 98°59'38.2"W, elev. 626 m) mainly on vegetation either with beating trays or by direct collecting. More information regarding this inventory and additional views of specimens can be found at www.unamfcaracnolab.com (Alvarez-Padilla Laboratory 2014).

Specimens were collected and stored in 96% ethanol. The female genitalia were dissected and digested following the protocol by Alvarez-Padilla and Hormiga (2008) and mounted using semi-permanent mounts (Coddington 1983). Photographs were taken with a Nikon DS-Fi2 camera, external anatomy images were taken using a Nikon SMZ1000 Stereomicroscope. Images of the cleared genitalia were taken with a Nikon E200 Microscope. Drawings were done with the respective drawing tubes for both microscopes. The images were captured using NIKON NIS ELEMENTS 4.0 software and multiple focal plane images were combined into montages using HELICON FOCUS 5.3.14. SEM samples were coated with platinum and images taken with a Zeiss EVO 55 SEM microscope. All measurements were performed using a micrometric ocular and are given in millimeters. High resolution versions of the images are available at www.unamfcaracnolab.com (Alvarez-Padilla Laboratory 2014). Mating plugs were documented by checking the available material from two collections: Museum of Comparative Zoology (MCZ) and the Arachnology lab from Facultad de Ciencias UNAM. A total of 127 specimens were examined documenting the presence of modified setae in both sexes, i.e., cymbium with broken and complete seta and epigynal genital openings either free or plugged (Table 1).

**Table 1.** Specimen strobilate setae presence or absence counts.

SEX	Total	Without seta	With seta	Left side	Right side	Both sides
MALE	47	18	29	24	24	19
FEMALE	80	38	42	25	20	3
Total	127	56	71	49	44	22

Abbreviations used in text and figures: (As) adjacent minor setae, (B) basis of seta, (BD) breakage disc, (bH) basal hematodocha, (CASC) California Academy of Sciences, (CO) copulatory opening, (dH) distal hematodocha, (E) embolus, (EF) epigynal flaps, (Fd) fertilization Ducts, (ip) irregular cuticular pigmentation, (M) thin cuticular layer between articles, (MCZ) Museum of Comparative Zoology at Harvard University, (RTA) retrolateral tibial apophysis, (Sp) spermathecae, (sSp) secondary spermathecae, (stS) strobilate seta.

## Systematics

### Maeota

Taxon classificationAnimaliaAraneaeSalticidae

Simon, 1901

#### Type species.

*Maeota dichrura* Simon, 1901

### Maeota setastrobilaris sp. n.

Taxon classificationAnimaliaAraneaeSalticidae

http://zoobank.org/2D91AD1F-47B1-438F-9E13-A12E621BAB93

Figures 1–7 , 8–15 , 16–23

#### Type materials.

**Holotype**: male from Jardín Escultórico Edward James, Xilitla, San Luis Potosí, Mexico 26–30 March 2012 (Alvarez-Padilla col.). **Allotype**: female with the same locality data and collected 10-15 June 2012 (Gonzáles-Contreras col.). Both specimen deposited at MZC, Harvard University.

#### Etymology.

The species epithet is a noun in apposition referring to the anatomy of the cymbial strobilate seta used as a mating plug, which resembles a strobilar gymnosperm cone.

#### Diagnosis.

*Maeota setastrobilaris* differs from *Maeota simoni* by the embolus coiled less than two times. It differs from *Maeota flava* and *Maeota dorsalis* by larger proximal tegular lobe and the finger shaped RTA extended ventrally (Fig. 13, 19). This species differs from *Maeota dichrura* by the shorter PLS and the absence of a lateral abdominal patch of enlarged setae in males. Unique features of *Maeota setastrobilaris* also include: epigynum copulatory duct openings covered by drop-shaped flaps (Fig. 23), embolus coiled 1.2 times and the cymbium dorso-basal edge with modified setae used as mating plugs.

#### Description.

*Male* total length 3.58 mm. Cephalothorax: length 1.83 mm, width 1.29 mm. Carapace dorsal surface dark-orange covered with scattered white scales and a glabrous longitudinal paler area posterior to the PLE. Carapace lateral surfaces and clypeus dark-orange with a reticulated pattern darker in color and concentrated towards the carapace edges (Fig. 18). Ocular anterior region: AME encircled with several scales, white at the center and orange at the sides. Ocular region 2/3 wider than long. PLE 0.5, PME 0.1 and ALE 0.5 times the diameter of AME. PME closer to PLE than ALE (Fig. 8). Chelicera paturon base dark-brown turning lighter distally, retromarginal tooth bifurcated. Endites pale-yellow distally, darker at their base and slightly longer than wide. Labium with same color pattern as the endites and triangular in shape. Sternum pale-yellow and longer than wide (Figs 11). Abdomen: surface background dark yellow covered with white or iridescent scales. Dorsal pattern with two brown longitudinal lines and a posterior broken pattern of dark chevrons (Fig. 8). Lateral surface almost covered by an additional dark reticulated thick line that extends towards the spinnerets. Ventral pattern with a dark gray rectangle that extends behind both sides of the epigastric furrow. Spinnerets gray and encircled with black pigmentation. PMS twice as thick as the PLS. PLS 2/3 longer than PMS with the distal segment darker in coloration and its ventral surface covered by thick black setae. Legs pale-yellow, I and II similar in size and smaller than III and IV. Leg IV the longest. Femora lighter than the other articles. Macroseta patterns based on voucher specimen JAM327. Femora with a 1-1-1 dorsal macroseta pattern. The distal spination has up to three subdorsal shorter macrosetae, two prolateral and one retrolateral. Patella with two subdorsal macrosetae, one on each side. Retrolateral tibia surfaces I to IV with six macrosetae distributed at 2-2-1-1 pattern. Tibia prolateral surfaces with the following macrosetae numbers and distributions: I, II 3-1-1-1; III-IV 3-2-1-1. Metatarsus prolateral surfaces: I 1-1-1-3; II 1-1-0-3; III-IV 2-0-0-3. Tarsi without macrosetae. Pedipalp: Tegulum with a proximal drop-shaped lobe and spermatic duct coils visible (Figs 14, 21). An additional sclerite present between the prolateral cymbial edge and the tegulum (Fig. 21 arrow). Embolus base circular, flat and attached to the embolus. Embolus heavily sclerotized, thick and coiling 1.2 times around embolus base edge (Fig. 21). Cymbium ventral surface notched distally near embolus, dorsal surface hirsute with four modified basal seta on the edge, the largest used as a mating plug (Fig. 19, stS). *Female* as in male except as noted. Total length 3.87 mm. Cephalotorax: length 1.62 mm, width 1.25 mm. Carapace lighter in coloration, reticular lateral pattern absent (Fig. 2). Abdomen: pattern as in male but lighter in coloration. Spinnerets: PLS 1.2 times longer than PMS. Legs: macroseta patterns based on voucher specimen JAM326. Retrolateral tibia surfaces: I-III 1-2-1-1; IV 2-1-1-1. Prolateral tibia surfaces: I, III-IV 1-2-1-1; II 1-1-1-1. Epigynum: with two comma-shaped, concentric cuticular flaps covering the genital opening entrance (Figs 6, 7, 20, 23). Spermathecae peanut-shaped with two lobes communicating by a thick channel, fertilization ducts tiny and located on the middle section of posterior lobes (Fig. 22).

**Figures 1–7.**
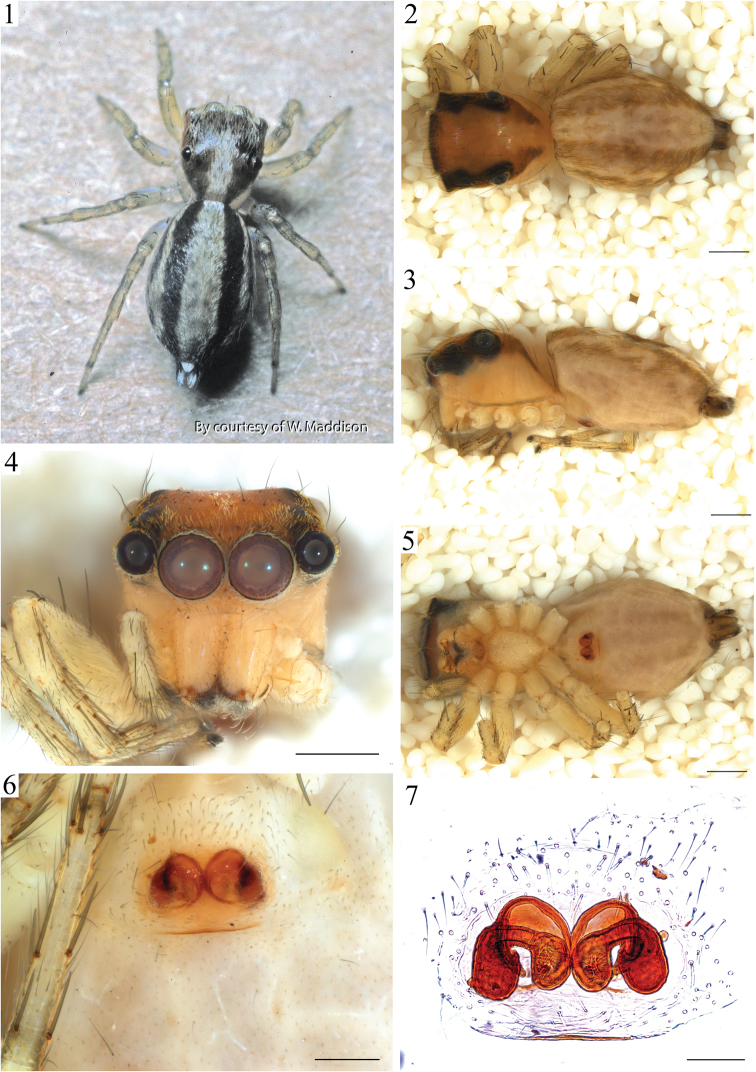
*Maeota setastrobilaris* female anatomy. **1** habitus live specimen **2** habitus of specimen in dorsal view **3** same lateral view **4** prosoma anterior view **5** habitus ventral view **6** epigynum ventral view **7** epigynum dorsal view (cleared). Scale bars 0.5 mm (**2–5**); 0.2 mm (**6**); and 0.1 mm (**7**).

**Figures 8–15.**
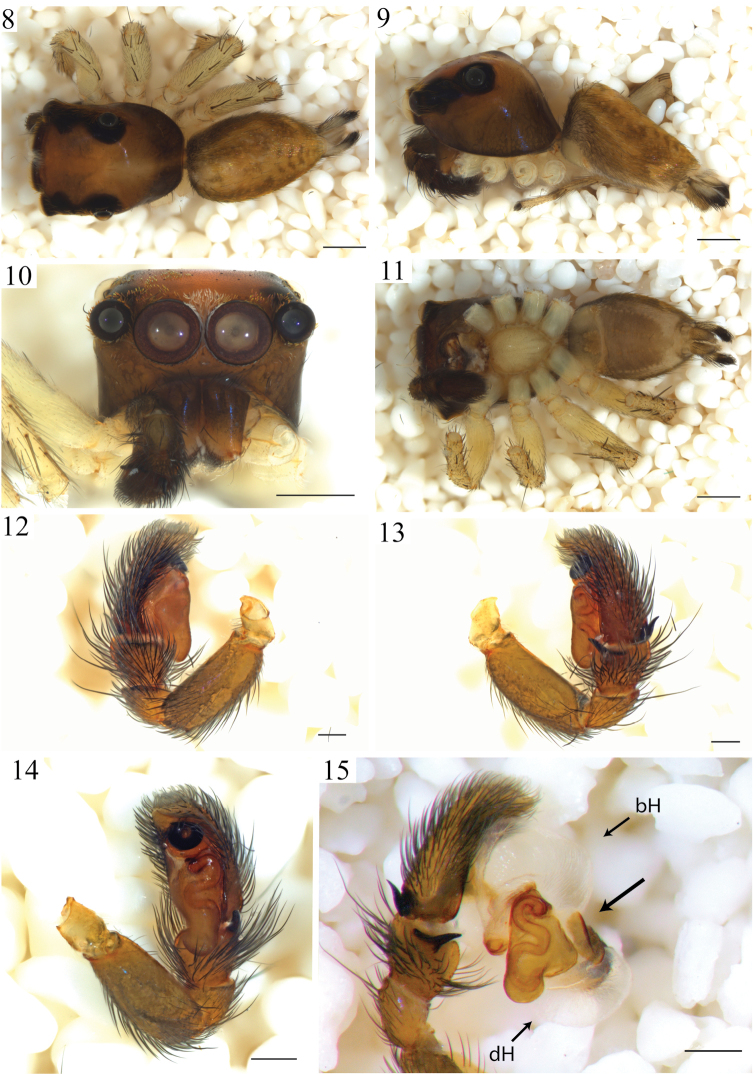
*Maeota setastrobilaris* male anatomy. **8** habitus dorsal view **9** same lateral view **10** prosoma anterior view **11** habitus ventral view **12** pedipalp prolateral view **13** same retrolateral view **14** same ventral view **15** pedipalp expanded retrolateral view. Scale bars: 0.5 mm (**8–11**); 0.2 mm (**12–15**). (bH, dH), basal and distal hematodochae, the larger arrow points to the conductor-like structure.

*Variation*: Male size: total length 2.76–3.58 mm, carapace 1.29–1.61 mm. Females total length 2.75–4.29 mm, carapace 1.20–1.70 mm. Spermathecal lobes vary considerably in orientation and the length of the channel. Several specimens presented asymmetric spermathecae. Flaps covering the copulatory ducts also vary in shape and in orientation relative to the middle longitudinal axis of the genital plate (Fig. 20). Specimen coloration varies from pale-yellow to dark-brown (Figs 1–5, 8–11).

**Figures 16–23.**
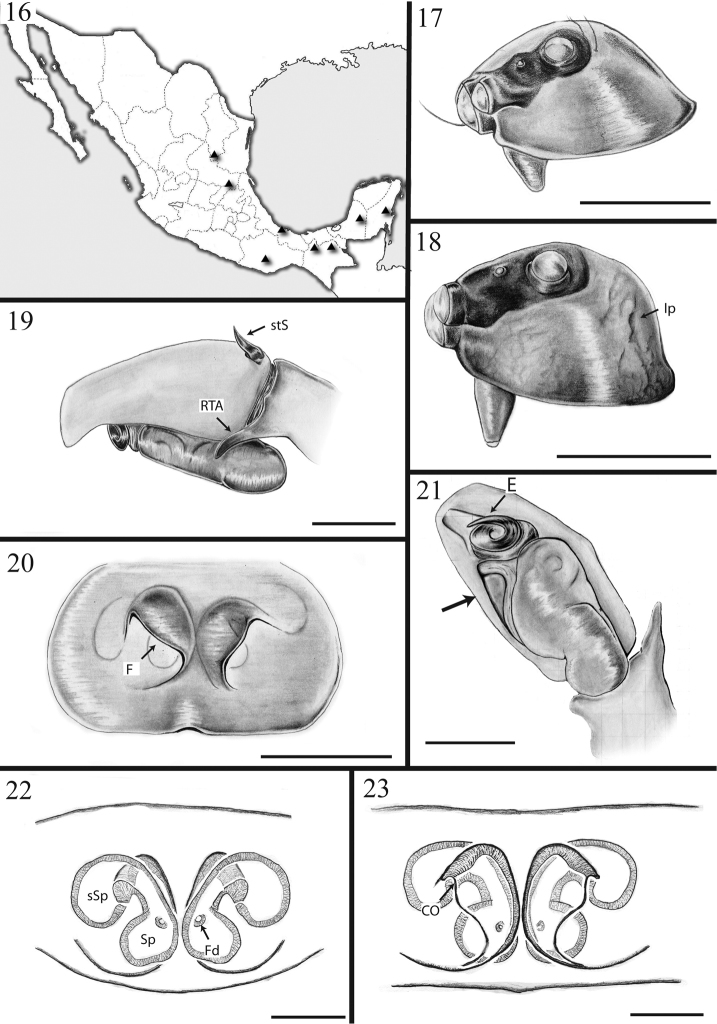
*Maeota setastrobilaris* distribution map and illustrations. 16, Distribution map. 17, female prosoma lateral view. 18, male prosoma lateral view. 19, male pedipalp retrolateral view. 20, epigynum ventral view. 21, male pedipalp ventral view 22 cleared epigynum dorsal view. 23, same ventral view. Scale bars 1 mm (**17, 18**); 0.2 mm (**19–21**); 0.1 mm (**22, 23**). (ip), irregular cuticular pigmentation, (stS) strobilate seta, (RTA) retrolateral tibial apophysis, (E) embolus, (EF) epigynal flaps, (Cd, Fd) copulatory and fertilization ducts, (sSp) secondary spermathecae, (Sp) spermathecae.

#### Distribution.

Mexico from the Eastern Cordillera to the Southwestern States. (Fig. 16).

*Records*: N = 139. **Mexico**: *Campeche*: Chicana ruins ca. 8 km W of Xpujil 18°32'N, 89°31'W 270 m. Jul. 14, 1983, W. Maddison col., 1♀, MCZ. *Chiapas*; 76 km S on road from Palenque to Ocosingo 17°01'N, 92°02'W 1377 m. Jul. 26–29, 1983, W. Maddison col., 1♀, 1♂, MCZ; Palenque ruins 17°29'N, 92°01'W 118 m. Jun. 02–11, 1983, W. Maddison & R.S. Anderson col., 4♀, 3♂, MCZ (paratypes); Jul. 31, 1983, W. Maddison col., 2♀, MCZ. *Nuevo León*: 29 E Linares along highway km 60 24°08'N, 99°08'W 197 m. Jun. 03–05, 1983, W. Maddison col., 1♂, MCZ. *Oaxaca*: 2 km S El Tule 17°02'N, 96°40'W 1868 m. 1983, W. Maddison & R.S. Anderson col., 1♀, MCZ. *Quintana Roo*: 31 NE Felipe Carrillo Puerto on highway km 307 19°48'N, 87°52'W 13 m. Jul. 17, 1983, W. Maddison & R.S. Anderson col., 1♂, MCZ. *San Luis Potosí*: Xilitla, Cueva de Salitre 21°23'N, 98°59'W 576 m. Jun. 13, 1983, W. Maddison col., 2♂, MCZ; Las Pozas (Jardín escultórico de Edward James) 21°23'50.9"N 98°59'38.2"W 626 m. Arachnology team col. Aug. 27–31, 2011, 13♀, 7♂; N. 14–18, 2011, 27♀, 11♂; Mar. 26–30, 2012, 25♀, 12♂; Jun. 10–15, 2012, 13♀, 12♂, Spider collection Arachnology Lab. Facultad de Ciencias. Supplementary images for paratypes deposited at CASC available at http://www.unamfcaracnolab.com with voucher and collection code numbers: N. 14–18, 2011, 1♀, JAM326 and CASENT 9051538; 1♂, JAM327 and CASENT 9051537. Additional specimens at CASC 1♀, 1♂, CASENT 9051536; 1♀, CASENT 9051539; 2♀, CASENT 9051540; 1♀, CASENT 9051541. *Tabasco*: 2.4 km. E Teapa, Grutas de Corona 17°33'N, 92°56'W 55 m. Jul. 7, 1983, W. Maddison col., 1♂, MCZ. *Veracruz*: Estacion Biología Tropical Los Tuxtlas near La Palma N of Catemaco 18°36'N, 95°07'W 366 m. 1983, W. Maddison & R.S. Anderson col., 1♀, MCZ.

#### Behavioral notes.

**Figures 24–30.**
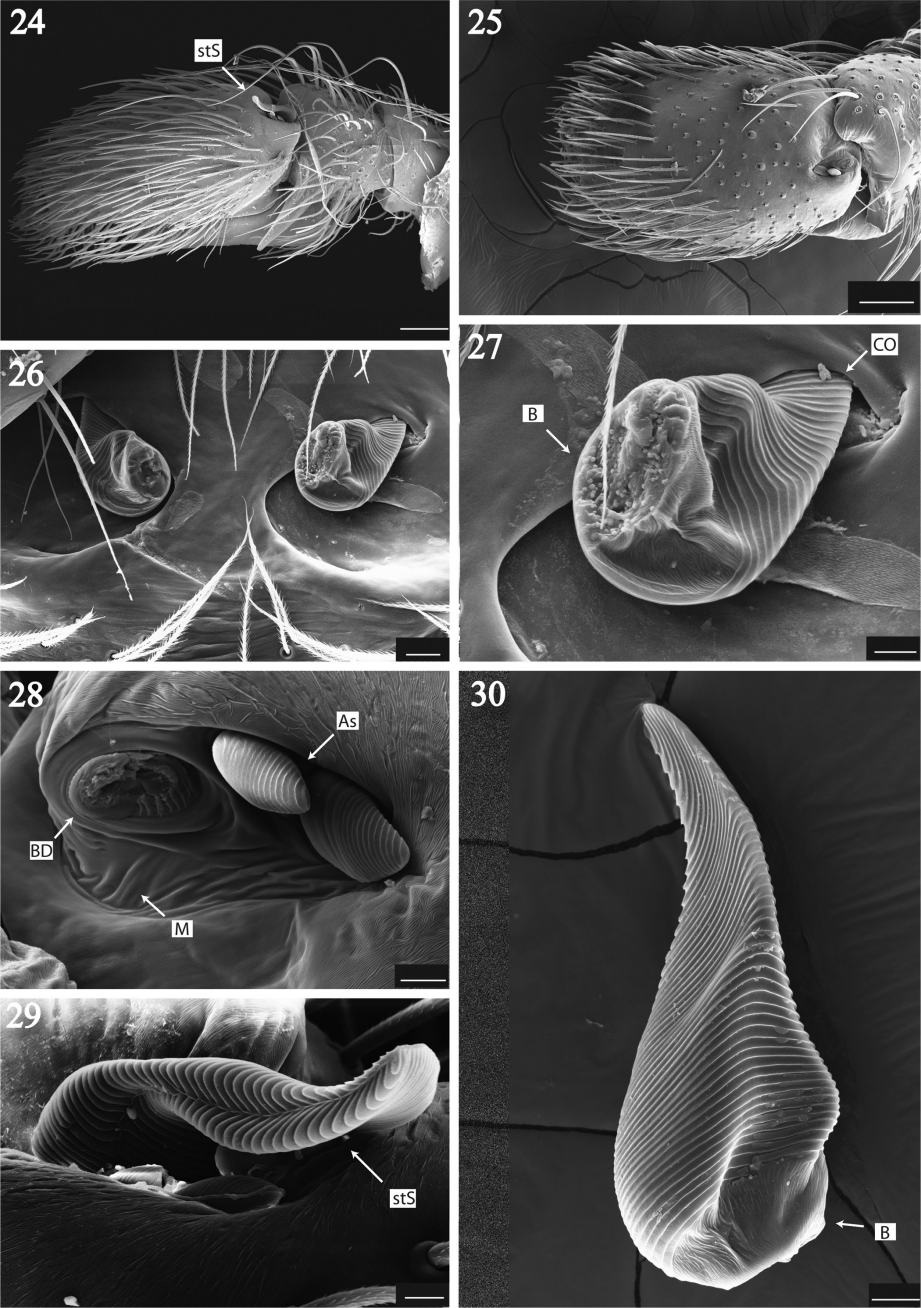
*Maeota setastrobilaris* mating plug SEM images. **24** cymbium at retrolateral view showing the seta **25** same view without seta **26** epigynal genital openings plugged by seta **27** close up of mating plug **28** strobilate seta brakeage disk **29** strobilate setae attached to cymbial membrane **30** detached strobilate seta. Scale bars 10 microns in all Figures. (B) basis of seta, (BD) breakage disc, (M) thin cuticular layer between articles, (As) adjacent minor setae, (stS) Strobilar seta, (CO) copulatory opening.

The dorsal base of cymbium presents a cluster of modified setae on its edge used during copulation as a mating plug. They are located over a pit between the membrane joining tibia-tarsus articles (Fig. 25: M). Inside the pit are three to four strobilate setae, the largest used as a mating plug while the others remain attached to the tibia-tarsus joint (Fig. 25). The largest of these strobilate setae is detached in several specimens and was found blocking the copulatory ducts entrance (Figs 24, 29: stS; 27, 28: CO). Evidence supporting the use of this strobilate seta as a mating plug lies in their rupture from a basal breakage disc (Fig. 28: BD) attached to weak setal basis (Figs 27, 30: B) and the texture that makes it difficult to mechanically extract the setae from the genital openings. The function of the smaller setae is unknown, but they could work either as pressure indicators controlling the detachment of the larger seta, or guiding it throughout the mating plug function. Total sample consisted of 127 specimens (Table 1) where 62% were females. Almost 50% of these females had the seta at least in one of their genital openings, while 60% of the males lost at least one seta from both pedipalps. Absence of both setae in males was higher than the presence of two setae in females with a ratio of 10:1. Accidental loss of setae from specimens after preservation is unlikely: exemplars collected 30 years ago that were examined in MCZ presented the structures in the same proportions as exemplars captured in 2011, suggesting that handling does not lead to accidental loss of these setae. The low proportion of females with both copulatory ducts plugged suggests a negative response to the insertion of a second seta.

## Supplementary Material

XML Treatment for Maeota

XML Treatment for Maeota setastrobilaris
